# Strong acids induce amyloid fibril formation of β_2_-microglobulin *via* an anion-binding mechanism

**DOI:** 10.1016/j.jbc.2021.101286

**Published:** 2021-10-07

**Authors:** Keiichi Yamaguchi, Kenshiro Hasuo, Masatomo So, Kensuke Ikenaka, Hideki Mochizuki, Yuji Goto

**Affiliations:** 1 Global Center for Medical Engineering and Informatics, Osaka University, Suita, Osaka, Japan; 2 Institute for Protein Research, Osaka University, Suita, Osaka, Japan; 3 Department of Neurology, Graduate School of Medicine, Osaka University, Suita, Osaka, Japan

**Keywords:** β_2_-microglobulin, strong acid, amyloid fibrils, solubility, supersaturation, β2m, β_2_-microglobulin, CD, circular dichroism, LS, light scattering, p*K*_a_, acid dissociation constant, pI, isotropic point, TCA, trichloroacetic acid, TEM, transmission electron microscopy, ThT, thioflavin T

## Abstract

Amyloid fibrils, crystal-like fibrillar aggregates of proteins associated with various amyloidoses, have the potential to propagate *via* a prion-like mechanism. Among known methodologies to dissolve preformed amyloid fibrils, acid treatment has been used with the expectation that the acids will degrade amyloid fibrils similar to acid inactivation of protein functions. Contrary to our expectation, treatment with strong acids, such as HCl or H_2_SO_4_, of β_2_-microglobulin (β2m) or insulin actually promoted amyloid fibril formation, proportionally to the concentration of acid used. A similar promotion was observed at pH 2.0 upon the addition of salts, such as NaCl or Na_2_SO_4_. Although trichloroacetic acid, another strong acid, promoted amyloid fibril formation of β2m, formic acid, a weak acid, did not, suggesting the dominant role of anions in promoting fibril formation of this protein. Comparison of the effects of acids and salts confirmed the critical role of anions, indicating that strong acids likely induce amyloid fibril formation *via* an anion-binding mechanism. The results suggest that although the addition of strong acids decreases pH, it is not useful for degrading amyloid fibrils, but rather induces or stabilizes amyloid fibrils *via* an anion-binding mechanism.

Amyloid fibrils are misfolded and self-assembled aggregates of proteins that are ∼10 nm in diameter and several micrometers in length and have a characteristic cross-β structure (1, 2, 3). The process of amyloid fibrils and oligomeric intermediates that result in amyloid fibrils may play crucial roles in the pathology of amyloid diseases (4, 5, 6). They are associated with more than 30 aberrant diseases, including Alzheimer's diseases, Parkinson's diseases, and dialysis-related amyloidosis (3, 7). In the case of dialysis-related amyloidosis (DRA), β_2_-microglobulin (β2m), consisting of 99 amino acid residues with one disulfide bond between Cys25 and Cys80, forms amyloid fibrils, which deposit at the joints and carpal tunnels in patients undertaking long-term hemodialysis (8, 9, 10). Although amyloid formation in patients occurs at a neutral pH, the acid denaturation promotes the amyloid formation, and thus extensive studies have been performed under acidic conditions as low as pH 2.0, revealing the basic mechanisms (11, 12, 13).

One of the most important properties clarified by amyloid formation at an acidic pH is its energetics. Amyloid fibrils are intermolecularly associated misfolded states driven by similar forces to those stabilizing the native states of proteins (hydrophobic interactions, hydrogen bonds, and van der Waals interactions). Proteins are acid-unfolded by the charge repulsion between positive charges. If the charge repulsions are shielded by salts through anion binding, they can form amyloid fibrils when the protein concentration is higher than the solubility (14, 15). In studies of protein folding at an acidic pH, it has been shown with various proteins that the shielding of positive charge repulsion by anion binding results in the formation of a molten globule state, a compact intermediate of protein folding with a significant amount of native-like secondary structures (16, 17, 18). Thus, while the formation of the molten globule state is an intramolecular reaction to respond to the decreased solubility of the unfolded state, the formation of amyloid fibrils is an intermolecular reaction to respond to the decreased solubility. Another important factor elucidated is the role of supersaturation (14, 19). Even if the concentration of an unfolded protein is higher than solubility, the breakdown of supersaturation by agitation or seeding is required to trigger the formation of amyloid fibrils. These factors should also be important at a neutral pH, although the reactions are more complicated because of the native structure that prevents amyloid formation (11, 20).

On the other hand, inhibiting the formation of amyloid fibrils and degrading preformed amyloid fibrils are important topics not only for preventing amyloidosis but also for reducing potential hazards associated with amyloid fibrils (21, 22, 23, 24, 25). Most recently, as for cleaning and disassembling potentially pathogenic amyloid-like assemblies of α-synuclein, tau, and Aβ 1 to 42, which can be adsorbed on nondisposable materials in laboratories, some commercial detergents and SDS (1% W/V) were shown to be the most effective (24, 25). Procedures used for decontamination and inactivation of amyloid pathogens tend to follow methodologies used for denaturing the native proteins. However, additives aimed to denature native proteins often induce amyloid formation because the solvent conditions to denature the native state tend to stabilize amyloid fibrils. In fact, although acid denaturation is one of the most common approaches for denaturing proteins, it has been shown that a combination of acid denaturation and increasing salt concentrations promotes amyloid formation of several proteins (14, 26). Moreover, it was shown more than 30 years ago that strong acids induce the molten globule state (15, 17, 18). The molten globule state has been suggested to be an intermediate of amyloid formation as well as that of protein folding (15). These results suggest the possibility of strong-acid-induced amyloid formation of proteins.

To assess the validity of using strong acids to denature amyloid fibrils, we examined the effects of strong acids on the amyloid formation of β2m. Although the effects of strong acid are less physiologically relevant, we observed strong-acid-induced amyloid formation, the extent of which was proportional to the concentration of acids and, thus, the decrease in pH. The results were understood on the basis of the anion-binding mechanism, suggesting that strong acids should be used carefully to avoid inducing anion-dependent amyloid formation. We summarize various conditions to stabilize or destabilize amyloid fibrils and their mechanisms in order to reduce the potential risks of amyloid fibrils.

## Results

### Amyloid formation of β2m at varying concentrations of NaCl and HCl

**Figure 1:**
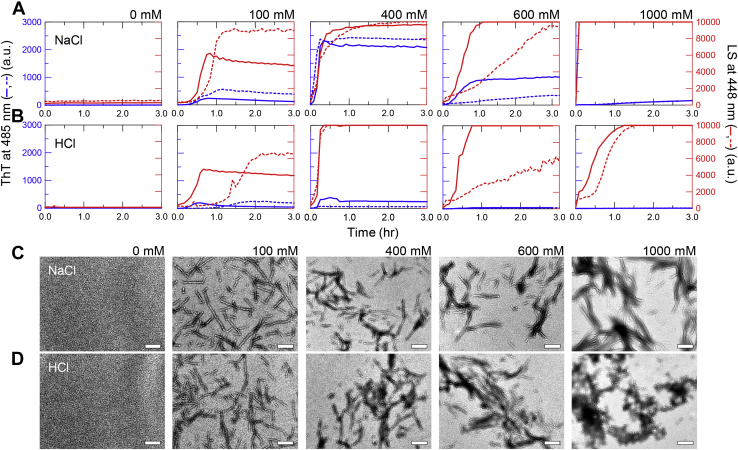
**Amyloid formation of β2m at varying concentrations of NaCl and HCl.***A* and *B*, kinetics of amyloid formation monitored by ThT fluorescence (*blue*) and LS (*red*) at varying concentrations of NaCl (*A*) and HCl (*B*) in the presence of 10 mM HCl under ultrasonication. *Solid lines* and *dashed lines* show separate measurements. The HCl concentrations indicated do not include the contribution of 10 mM HCl. *C* and *D*, EM images of amyloid fibrils or amorphous aggregates at varying concentrations of NaCl (*C*) and HCl (*D*). Scale bars are 200 nm.

To investigate the effects of NaCl and HCl, we performed the amyloid formation of β2m at varying concentrations of NaCl and HCl in the presence of 10 mM HCl under ultrasonication (Fig. 1, *A* and *B*). We simultaneously measured thioflavin T (ThT) fluorescence and light scattering (LS); ThT fluorescence and LS monitored the amyloid fibrils and total amount of aggregates including both amyloid fibrils and amorphous aggregates, respectively (14, 27, 28). As a control of acid unfolding without salts or additional acids, the solutions contained 10 mM HCl (pH approximately 2.0), where both ThT fluorescence and LS did not increase (Fig. 1, *A* and *B*; 0 mM). According to the solubility and supersaturation mechanism, the solubility of β2m was higher than 25 μM β2m in 10 mM HCl.

**Figure 2:**
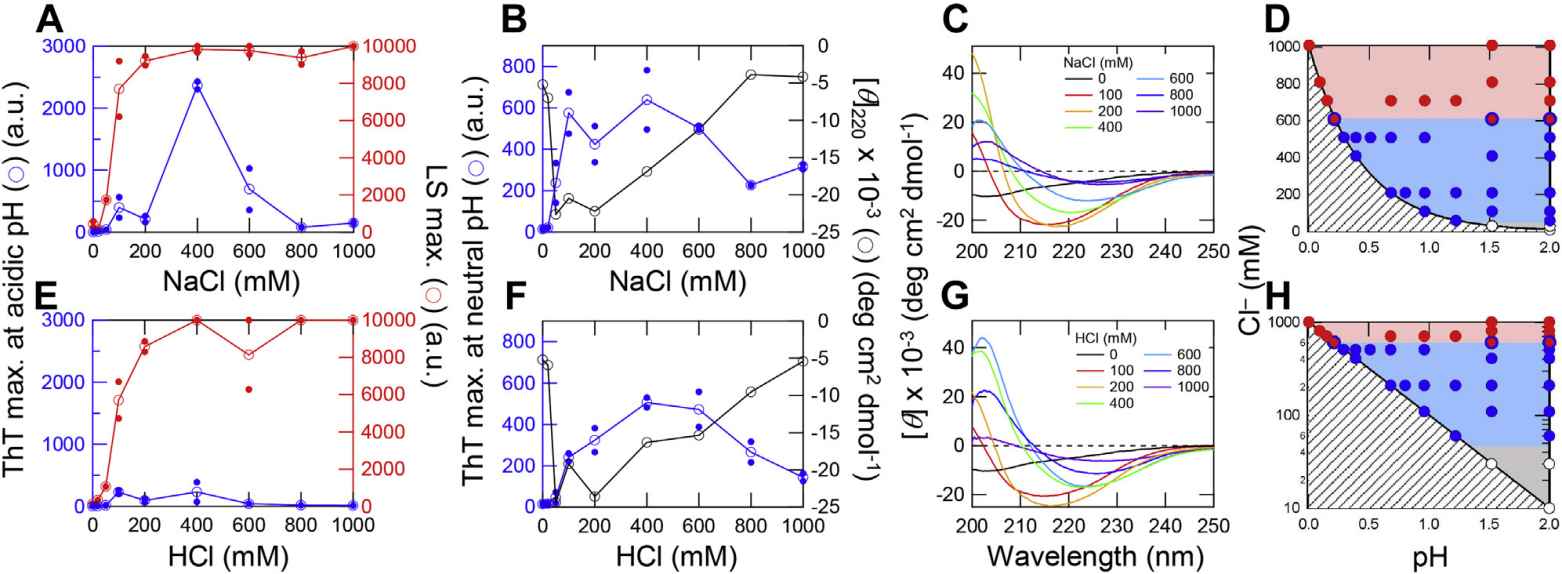
**Amyloid formation and phase diagram at varying concentrations of NaCl and HCl.***A* and *E*, maximum values of ThT fluorescence (*blue*) and LS (*red*) at varying concentrations of NaCl (*A*) and HCl (*E*) in the presence of 10 mM HCl. *Open symbols* indicate the average values and *small closed symbols* indicate the observed data; when only one data point was available, they are overlapped. The concentrations of NaCl and HCl were plotted after subtraction of the 10 mM HCl component. *B* and *F*, maximum values of ThT fluorescence under neutral conditions (*blue*) and CD ellipticities at 220 nm (*black*). *C* and *G*, CD spectra obtained after amyloid formation at varying concentrations of NaCl (*C*) and HCl (*G*). The CD spectrum at 0 mM HCl (*G*) was taken from 0 mM NaCl (*C*). *D* and *H*, phase diagram of HCl- or NaCl-induced amyloid formation plotted by the conformational states. Monomer and amyloid fibrils and amorphous aggregates are represented by *white* and *blue* and *red circles*, respectively. The Cl^−^ concentration on the vertical axis was plotted either linearly (*D*) or in a log scale (*H*). The *shaded area* is prohibited owing to an increase in the concentration of Cl^−^ with a decrease in the pH of the solution. To construct the phase diagram, Cl^−^ concentration-dependent amyloid formations at 200, 500, and 700 mM Cl^−^ in 10 mM HCl were measured (Figs. S4, *A*–*C* and S5, *A*–*C*), and NaCl concentration-dependent amyloid formations at pH 1.5 were measured (Figs. S4*D* and S5*D*).

At 100 mM NaCl, the ThT fluorescence and LS increased slightly after a lag time of 30 min (Fig. 1*A*). At 400 mM NaCl, both the ThT fluorescence and LS increased markedly with a sigmoidal pattern. At 600 mM NaCl, ThT fluorescence increased moderately although LS increased markedly. At 1000 mM NaCl, only LS increased within the dead time (approximately 10 s) of the measurements to a level beyond the instrumental detection, indicating the extensive formation of amorphous aggregates. Transmission electron microscopy (TEM) observation showed the formation of rigid amyloid fibrils at 100 to 400 mM NaCl (Fig. 1*C*), and the formation of laterally associated fibrils as well as amorphous aggregates at 600 to 1000 mM NaCl, although high concentrations of NaCl or HCl prevented clear TEM images. The maximum values of ThT fluorescence and LS plotted against the NaCl concentration (Fig. 2*A*) showed that the optimum concentration for amyloid formation was around 400 mM NaCl, and the associated fibrils and amorphous aggregates were formed at the higher NaCl concentrations, as previously observed (14, 26, 28, 29).

In the presence of varying concentrations of HCl, LS significantly increased with an increase in the HCl concentration, although ThT increased slightly at 100 mM HCl (Figs. 1*B* and 2*E*). The reproducibility of lag time was not high at 100 mM HCl, probably depending on the strength of the ultrasonication. However, the TEM observation showed that β2m formed rigid fibrils at 100 to 400 mM HCl and mixtures of laterally associated fibrils and amorphous aggregates at 600 to 1000 mM NaCl (Fig. 1*D*), as observed for the NaCl-dependent amyloid formation (Fig. 1*C*). ThT has a titratable tertiary amine group with a p*K*_a_ value of 1.2 (30) (Fig. S1). Thus, the lack of ThT fluorescence even with the formation of aggregates of fibrillar morphology suggested that the acid quenching prevented the detection by ThT fluorescence. Therefore, we increased the pH by diluting the acid- and ultrasonication-treated β2m solutions by 100 times with 50 mM sodium phosphate buffer at pH 7.5 containing 5 μM ThT. We then immediately measured the ThT fluorescence, because the amyloid fibrils formed at an acidic pH tended to dissolve at a neutral pH (31, 32) (Fig. S2, *A* and *B*).

Using the assay at pH 7.5, we observed an increase in ThT fluorescence with the optimum at 400 mM HCl (Fig. 2*F*), which was comparable to that of the NaCl-dependent amyloid formation at pH 2.0 (Fig. 2*B*). We also confirmed the formation of amyloid fibrils using circular dichroism (CD) spectroscopy. The far-UV CD spectra indicated that the CD ellipticities at 220 nm decreased with increasing salt concentration starting from 100 mM, which did not correlate with their ThT results. The far-UV CD spectra showed minima at 210 to 215 nm and large negative ellipticities (approximately −20,000 deg cm^2^ dmol^−1^) at 100 mM NaCl or HCl, suggesting that the correct CD spectra could not be obtained because of serious aggregation. Since the ThT fluorescence indicates the total amount of amyloid fibrils more reliably than the CD, we assumed that amyloid fibrils were formed with an optimal concentration of 400 mM for both NaCl and HCl. At NaCl or HCl concentrations higher than 400 mM, the CD spectra changed to those with a negative peak at around 230 nm and with reduced intensity, suggesting an increasing amount of amorphous aggregates, consistent with the ThT, LS, and TEM results. We analyzed the kinetics data using the Finke–Watzky (F-W) two-step kinetics model (33, 34) and included the related discussion in Figure S3.

We then constructed a phase diagram for the HCl- or NaCl-induced amyloid formation by plotting the conformational states (monomer, amyloid, and amorphous) against the solution pH on the horizontal axis and Cl^−^ concentration on the vertical axis. To focus on the monomeric state existing at low anion concentrations, the vertical axis was plotted in either linear (Fig. 2*D*) or log (Fig. 2*H*) scales. To construct the phase diagram, we also examined Cl^−^ concentration-dependent amyloid formations at 200, 500, and 700 mM Cl^−^ in addition to 10 mM HCl (Fig. S4, *A*–*C* and S5, *A*–*C*), where the concentrations of NaCl and HCl were adjusted to maintain constant Cl^−^ concentrations. Moreover, we performed NaCl concentration-dependent amyloid formation at pH 1.5 (Figs. S4*D* and S5*D*). The concentrations of NaCl and HCl were plotted after subtraction of 10 mM HCl in Figure 2, *A* and *E*, because the same subtraction should be done to compare the effects of other salts and acids. On the other hand, in the pH- and Cl^−^-dependent phase diagram, the contribution of 10 mM HCl was included to show the exact Cl^−^ concentration. The conformational states (monomeric unfolded, amyloid, and amorphous states) were assessed mainly on the basis of ThT fluorescence and LS. The unfolded monomers have neither ThT fluorescence nor LS, amyloid fibrils have both ThT fluorescence and LS, and amorphous aggregates have only LS. The results of TEM and CD measurements were also considered. It can be noted that decreasing the pH by HCl below 2.0 exponentially increases the concentration of Cl^−^ anions as well as that of protons by the equation: [Cl^−^] = [H^+^] = 10^−pH^ (mol/l), so that the shaded area in the phase diagram is not allowed.

**Figure 3:**
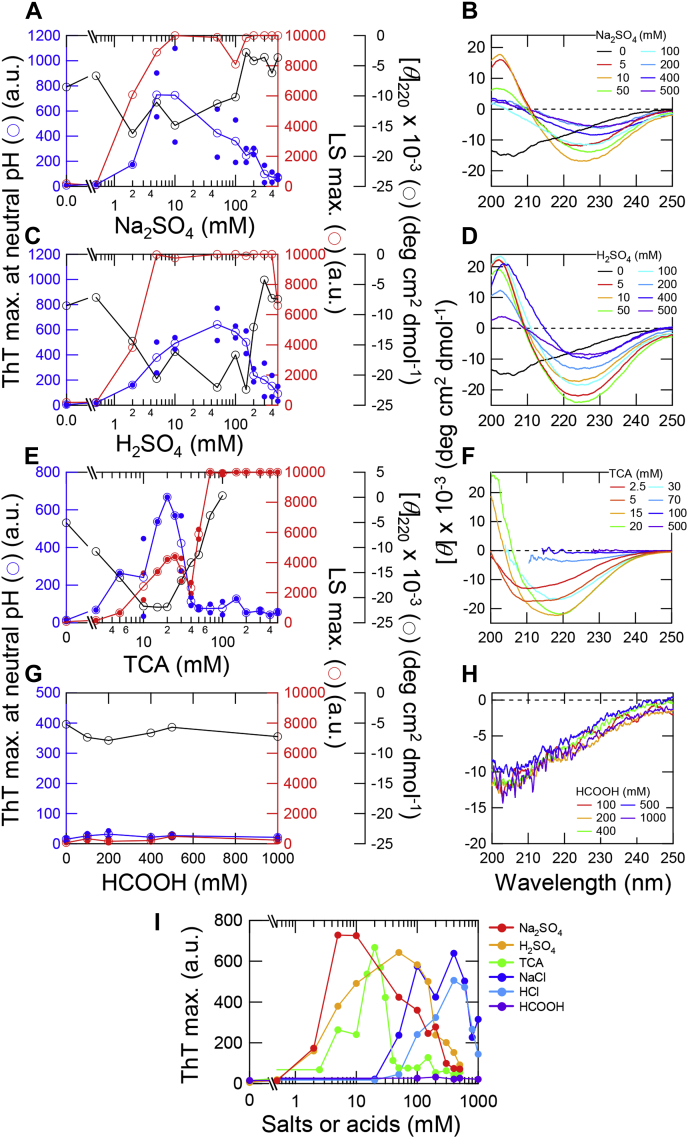
**Amyloid formation of β2m at varying concentrations of Na**_**2**_**SO**_**4**_**, H**_**2**_**SO**_**4**_**, TCA, or formic acid under ultrasonication.***A* and *C*, maximum values of ThT fluorescence at a neutral pH (*blue*) and LS (*red*), and CD ellipticities at 220 nm (*black*) at varying concentrations of Na_2_SO_4_ (*A*) and H_2_SO_4_ (*C*) in the presence of 10 mM HCl. ThT fluorescence was measured under neutral pH conditions. *Open symbols* indicate the average values and *small closed symbols* indicate the observed data; when only one data point was available, they are overlapped. *B* and *D*, CD spectra obtained after amyloid formation at varying concentrations of Na_2_SO_4_ (*B*) and H_2_SO_4_ (*D*). The CD spectrum at 0 mM H_2_SO_4_ (*D*) was taken from that at 0 mM Na_2_SO_4_ (*B*). *E* and *G*, maximum values of ThT fluorescence at a neutral pH (*blue*) and LS (*red*), and CD ellipticities at 220 nm (*black*) at varying concentrations of TCA (*E*) and formic acid (*G*) in the presence of 10 mM HCl. *F* and *H*, CD spectra obtained after amyloid formation at varying concentrations of TCA (*F*) and formic acid (*H*). TCA has large absorption in the far-UV region. The regions including noise were removed from the CD spectra. *I*, maximum values of ThT fluorescence against the concentrations of salts or acids. The HCl concentration does not include the contribution of 10 mM HCl. Kinetics of amyloid formation at varying concentrations of Na_2_SO_4_, H_2_SO_4_, TCA, and formic acid were shown in Figure S7.

The phase diagram showed that, when the Cl^−^ concentration increased at a constant pH below 2, the conformation of β2m changed from an unfolded monomer at 0 mM Cl^−^ (Fig. 2, *D* and *H*; white circles), to an amyloid fibril at 100 to 600 mM Cl^−^ (Fig. 2, *D* and *H*; blue circles) and amorphous aggregate at 600 to 1000 mM Cl^−^ (Fig. 2, *D* and *H*; red circles). In contrast, an increase in HCl in the absence of NaCl followed a boundary line between the accessible and prohibited areas (Fig. 2, *D* and *H*; shaded area), showing the dominance of the unfolded monomer at 0 to 100 mM Cl^−^, amyloid fibril at 100 to 600 mM Cl^−^, and amorphous aggregate at 600 to 1000 mM Cl^−^. In fact, the NaCl- and HCl-dependent transitions monitored by ThT fluorescence agreed well, confirming the dominant role of Cl^−^ anions (Fig. 3*I*).

### Amyloid formation of β2m at varying concentrations of Na_2_SO_4_ and H_2_SO_4_

To compare the effects of Na_2_SO_4_ and H_2_SO_4_, we first checked the acid hydrolysis of β2m at 37 °C in the presence of 500 mM H_2_SO_4_ using HPLC and ESI-Mass (Fig. S6). The proteins might be chemically decomposed at higher concentrations of H_2_SO_4_ (>0.5 M) at 90 °C (35). We observed no degradation of β2m during an incubation of 3 h at 37 °C under ultrasonication, the conditions employed in this study, but β2m slightly degraded after an incubation of 6 h. We also examined the stability of ThT compound in 1.0 M HCl or 0.5 M H_2_SO_4_, 37 °C (Fig. S1, *D*–*F*) and confirmed that ThT compound did not degrade for at least 19 h under the acidic conditions used here.

As the result of amyloid formation at varying concentrations of Na_2_SO_4_ and H_2_SO_4_, we observed increases in ThT fluorescence at concentrations higher than ∼5 mM Na_2_SO_4_ (Figs. 3*A* and S7*A*) and ∼5 mM H_2_SO_4_ (Figs. 3*C* and S7*B*). The amyloid formation of β2m dependent on Na_2_SO_4_ was previously reported to occur at approximately 5 mM under acidic conditions (26). At the higher concentrations of Na_2_SO_4_, we observed a marked increase in LS but not ThT fluorescence, indicating the formation of amorphous aggregates. The same was true for H_2_SO_4_ even though we assessed ThT fluorescence at a neutral pH. The far-UV CD spectra showed a β-sheet-rich conformation with minima at 218 to 220 nm upon the formation of amyloid fibrils induced by Na_2_SO_4_ (Fig. 3*B*) or H_2_SO_4_ (Fig. 3*D*). TEM observation showed that amyloid fibrils and amorphous aggregates were formed at 10 to 100 and 200 to 500 mM Na_2_SO_4_, respectively (Fig. S8*A*) and at 10 to 100 and 200 to 500 mM H_2_SO_4_, respectively (Fig. S8*B*). Thus, amyloid fibril formation was induced at moderate concentrations of H_2_SO_4_ as well as Na_2_SO_4_.

### Amyloid formation of β2m at varying concentrations of TCA, but not formic acid

We also examined the effects of trichloroacetic acid (TCA) and formic acid on amyloid formation. In research on prion disease, treatment with formic acid at a concentration higher than >60% for longer than 1 h or with TCA at a concentration higher than 3 M for longer than 2 h both at room temperature was employed for inactivation of infectious prions (21). TCA has been traditionally used for protein precipitation coupled with denaturation (36).

We found that TCA at concentrations of approximately 20 mM markedly induced amyloid fibril formation (Figs. 3*E* and S7*C*). The far-UV CD spectra at 10 to 20 mM TCA exhibited β-sheet-rich conformation with a minimum at around 210 to 215 nm (Fig. 3*F*), where rigid amyloid fibrils were observed by TEM (Fig. S8*C*). At higher concentrations of TCA, ThT fluorescence did not increase (Fig. 3*E*) and the amorphous aggregates were prevalent when observed by TEM (Fig. S8*C*). Thus, in the TCA precipitation, amyloid fibrils populated under a narrow region above solubility, and amorphous aggregates were prevalent at higher TCA concentrations.

In contrast, formic acid did not induce amyloid fibrils (Figs. 3*G* and S7*D*), and the far-UV CD spectra revealed a disordered conformation at all concentrations examined (Fig. 3*H*). TEM observation showed that, in the presence of formic acid, β2m formed small particles, which are unlikely to be amyloid fibrils (Fig. S8*D*).

Here, an acidic p*K*_a_ value determines the extent of ionization to produce anions. HCl is a strong acid with a p*K*_a_ value of −8, leading to complete dissociation to H^+^ and Cl^−^ under experimental conditions below pH 2.0. Although H_2_SO_4_ is a strong acid, it has two p*K*_a_ values at −5 and 1.99, making a net charge of −1.5 at pH 2.0. TCA is a strong acid with a p*K*_a_ value of 0.66 with approximately 80% dissociated at pH 2.0. On the other hand, formic acid is a weak acid with a p*K*_a_ value of 3.75, producing no anion under the experimental conditions at pH 2.0. These p*K*_a_ values suggested that anion concentrations and not the pH are important for determining acid-dependent amyloid formation.

We plotted the ThT fluorescence at a neutral pH, representing the amount of amyloid fibrils, against the concentrations of acids or salts (Fig. 3*I*). The plots indicated two important points. First, the salt- and acid transitions agreed fairly well, confirming the critical role of anions in determining the acid-induced amyloid formation. Second, the effectiveness of various anions differs notably in the order: sulfate^1.5−^ > TCA^−^ > Cl^−^. This order is consistent with the electroselectivity series rather than Hofmeister series because the trichloroacetate anion is more effective than the chloride anion. Moreover, this order is consistent with the effectiveness of anions in stabilizing the acidic molten globule states (18). It has been established that the salt-dependent promotion of amyloid formation of β2m is caused by the anion binding effects by examining the effects of a larger number of anions (26). We conclude that strong-acid-induced amyloid formation of β2m occurs by the same mechanism as the anion-dependent amyloid formation at pH 2.0.

### HCl-dependent amyloid formation of insulin

**Figure 4:**
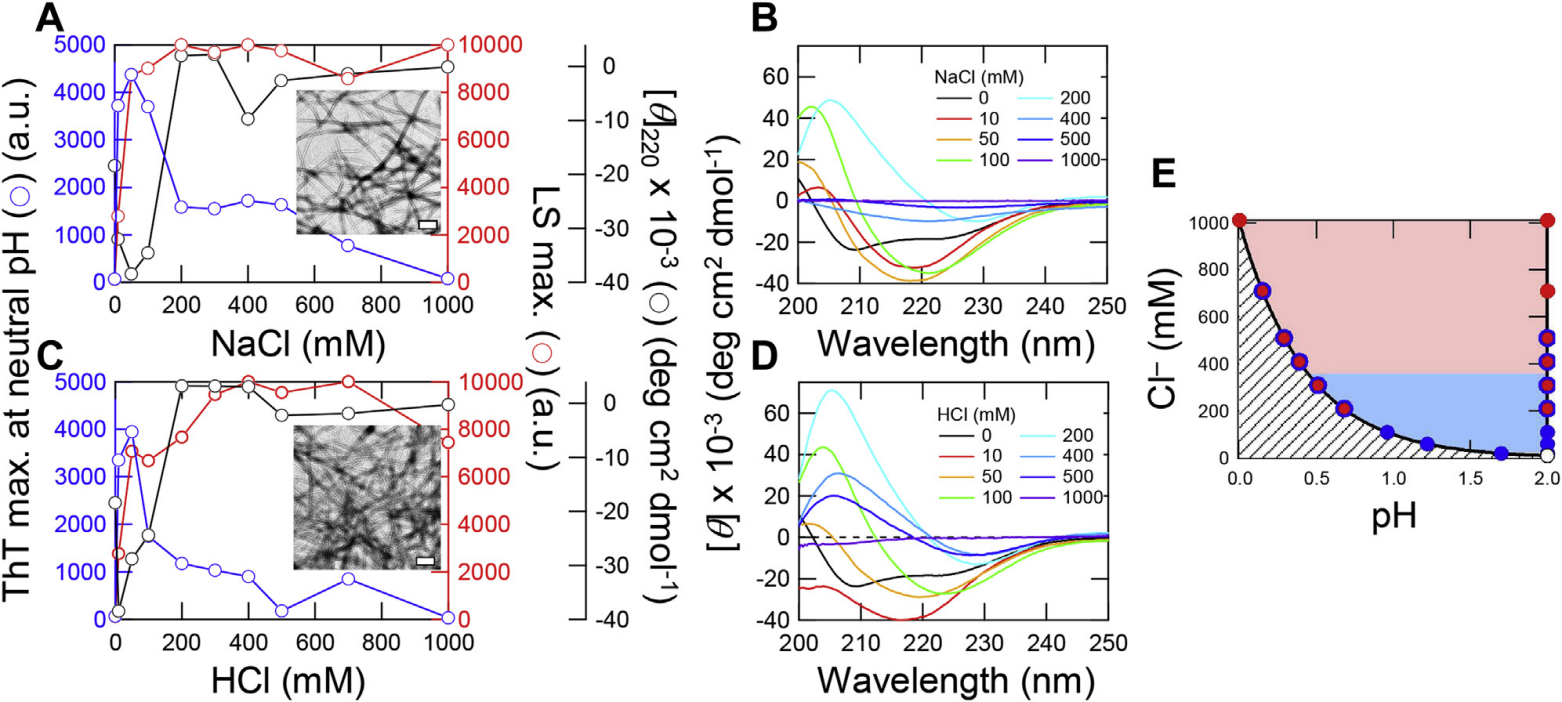
**Amyloid formation of insulin at varying concentrations of NaCl or HCl under ultrasonication.***A* and *C*, maximum values of ThT fluorescence at a neutral pH (*blue*) and LS (*red*), and CD ellipticities at 220 nm (*black*) at varying concentrations of NaCl (*A*) and HCl (*C*) in the presence of 10 mM HCl. ThT fluorescence was measured under neutral pH conditions. The concentrations of NaCl and HCl were plotted after subtraction of the 10 mM HCl component. The *insets* show EM images of amyloid fibrils at 50 mM NaCl (*A*) and HCl (*C*). Scale bars are 200 nm. *B* and *D*, CD spectra obtained after amyloid formation at varying concentrations of NaCl (*B*) and HCl (*D*). *E*, phase diagram of HCl- or NaCl-induced amyloid formation of insulin plotted by the conformational states. Monomer and amyloid fibrils and amorphous aggregates are represented by *white* and *blue* and *red circles*, respectively. The *shaded area* is prohibited owing to the increase in the concentration of Cl^−^ with a decrease in the pH of the solution. Kinetics of amyloid formation at varying concentrations of NaCl and HCl were shown in Figure S9.

To explore the generality of acid-induced amyloid formation, we investigated the amyloid formation of insulin at varying concentrations of NaCl and HCl. Insulin formed amyloid fibrils at NaCl concentrations higher than 50 mM at 10 mM HCl (pH 2.0) under ultrasonication (Figs. 4*A* and S9*A*). However, at higher concentrations of NaCl, ThT fluorescence decreased, suggesting the increasing formation of amorphous aggregates. The far UV-CD spectra showed an α-helical conformation at 0 mM NaCl (Fig. 4*B*), a β-sheet-rich conformation between 10 and 100 mM NaCl, and disordered conformations above 200 mM NaCl. It is likely that, as was the case with β2m, we could not accurately measure CD spectra at high concentrations of NaCl because of the formation of large aggregates.

A similar conformational transition was observed with HCl (Figs. 4*C* and S9*B*). Amyloid formation monitored by ThT fluorescence was observed at 10 to 100 mM HCl, where the ThT assay was performed at pH 7.5 (Fig. 4*C*). Above 100 mM HCl, ThT fluorescence did not increase even when monitored at pH 7.5, indicating the dominance of amorphous aggregates. The far UV-CD spectra showed the formation of amyloid fibrils at 10 to 100 mM HCl and amorphous aggregates above 200 mM HCl (Fig. 4*D*), consistent with the ThT and LS measurements. TEM observation also showed that amyloid fibrils were formed at 50 mM NaCl or HCl (Fig. 4, *A* and *C*; insets), and both amyloid fibrils and amorphous aggregates were formed at 200 to 500 mM NaCl or HCl (Fig. S8, *E* and *F*). Amorphous aggregates were formed at 1000 mM NaCl or HCl.

The phase diagram of insulin for amyloid formation against the pH of the solution and Cl^−^ concentrations was constructed in a similar way to that of β2m mainly based on ThT fluorescence and LS. The phase diagram showed that insulin formed amyloid fibrils at concentrations of 10 to 100 mM Cl^−^ either from NaCl or HCl, and at higher concentrations of Cl^−^, amorphous aggregates were also formed (Fig. 4*E*). At 1000 mM Cl^−^, amorphous aggregates dominated. These results were in agreement with those of β2m, suggesting the generality of acid-induced amyloid formation.

### Acid-induced molten globule conformation of β2m

**Figure 5:**
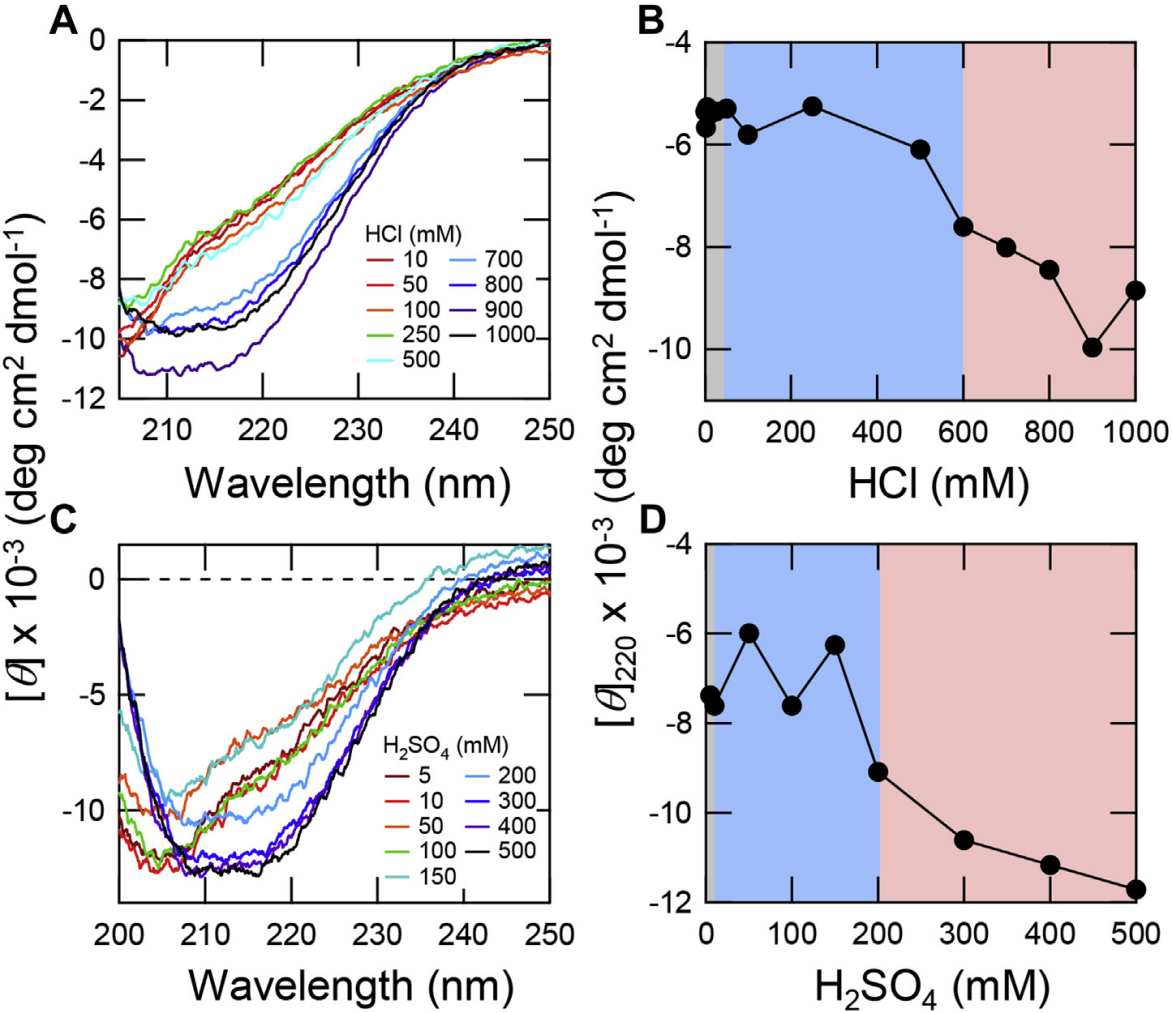
**The formation of a molten globule under acidic conditions.***A* and *C*, CD spectra of β2m obtained before amyloid formation at varying concentrations of HCl (*A*) and H_2_SO_4_ (*C*) without ultrasonication. *B* and *D*, CD ellipticities at 220 nm at varying concentrations of HCl (*B*) and H_2_SO_4_ (*D*). The concentration ranges to form monomer, amyloid fibrils, and amorphous aggregates are shown in *gray*, *light blue*, and *pink*, respectively.

It was reported that β2m exhibited a monomeric conformational transition from the highly unfolded state to a compact intermediate state upon the addition of salts, and such a transition is linked to the promotion of amyloid fibril formation (29, 37). Here, without ultrasonication to suppress amyloid formation, we observed a conformational change of β2m from an acid-unfolded conformation to a molten globule state at 600 to 1000 mM HCl (Fig. 5*A*). H_2_SO_4_ also induced the conformational change from an unfolded to a molten globule conformation with an increase in the H_2_SO_4_ concentration (Fig. 5*C*). Intriguingly, the concentration ranges to form the molten globule in the presence of HCl (Fig. 5*B*) or H_2_SO_4_ (Fig. 5*D*) agreed with those to form amorphous aggregates in the presence of HCl (Fig. 2) or H_2_SO_4_ (Fig. 3*C*), respectively, under ultrasonication.

It should be noted that insulin did not show the molten globule conformation depending on the concentration of acid, since it showed the native-like conformation even under acidic pH conditions. Thus, anions are crucial for the formation of amyloid fibrils and partially folded molten globule state under acidic conditions by inter- and intramolecular interactions, respectively.

### Effects of strong acids on preformed amyloid fibrils

Finally, we examined possible destabilization of the preformed amyloid fibrils by acid treatment. We first prepared β2m amyloid fibrils at pH 2.0 or 7.0, and then they were transferred to strongly acidic solutions such as 1.0 M HCl or 0.5 M H_2_SO_4_. However, the preformed amyloid fibrils prepared at both pH 2.0 (Fig. S2, *C* and *E*) and 7.0 (Fig. S2, *D* and *F*) were hardly destabilized by strong acid treatments. It is possible that preformed amyloid fibrils were further stabilized by the anion-binding mechanism.

## Discussion

### Mechanism of acid-induced amyloid fibril formation

**Figure 6:**
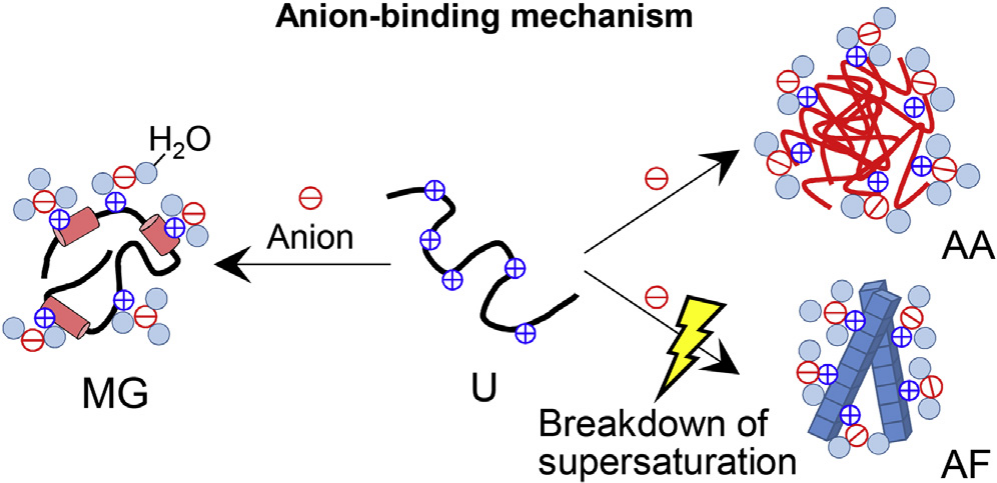
**Mechanisms of strong-acid-induced amyloid formation.** An increase in the anion concentration induces the intermolecular interaction leading to the formation of amyloid fibrils (AF) or amorphous aggregates (AA) and also induces the intramolecular interaction leading to the formation of molten globule (MG). Amyloid fibrils were formed with the breakdown of supersaturation under ultrasonication.

Previously, during the study of the acidic molten globule state of proteins, Goto *et al.* (15, 17, 18) found that strong acids as well as salts stabilize the molten globule state of proteins (*e.g.*, β-lactamase, cytochrome *c*, and apomyoglobin). Although salt effects occur at a pH of around 2.0, the effects of acids occur increasingly with a decrease in the pH below 2.0. The acid- and salt-induced transitions agreed very well when plotted against the anion concentrations, showing that anions are responsible for the formation of the molten globule states. The pH- and anion concentration-dependent phase diagram revealed how the decrease in the pH has the same effects as the increase in the salt concentration at a constant pH of around 2.0 by the relation: [Cl^−^] = [H^+^] = 10^−pH^ (mol/l). Moreover, comparison of various salts and acids showed that the effectiveness of anions followed the electroselectivity series, confirming that anion effects arise from the counter anion binding to the positively charged unfolded proteins. In other words, shielding of charge repulsions results in the manifestation of hydrophobic interactions, leading to formation of the molten globule states (Fig. 6).

Because the amyloid fibrils are stabilized dominantly by the same interactions as those stabilizing the native or molten globule states, it is not surprising that the conditions stabilizing the molten globule states also promoted the formation of amyloid fibrils. Amyloid fibrils are intermolecularly misfolded states formed upon breaking the supersaturation barrier (Fig. 6). Thus, under acidic conditions where the tight packing of side chains required for the native states cannot be achieved, proteins may form amyloid fibrils instead of molten globule states under the conditions where the protein concentration is higher than solubility. When amyloid fibrils are regarded as crystal-like precipitates of denatured proteins, amorphous aggregates are presumed to populate under conditions where the driving forces of precipitation are too strong to retain amyloid fibrils (Fig. 6) (19, 38). This viewpoint enables a more comprehensive understanding of aggregation in terms of solubility, supersaturation (or supercooling), and competition between the two types of aggregates (11, 20).

It is not clearly known if the molten globule sates are located on the pathway to amyloid formation or it is an off-pathway product competing with the formation of amyloid fibrils. In our case, however, the acid concentrations stabilizing the molten globule states (>600 mM HCl or >200 mM H_2_SO_4_, Fig. 5) were higher than those promoting amyloid fibril formation (>50 mM HCl or >5 mM H_2_SO_4_, Figs. 2 and 3). Those high anion concentrations stabilizing the molten globule states tended to lead to amorphous aggregates upon prolonged incubation. These results suggest that the molten globule states and related intermediate states including oligomers are off-pathway products of amyloid formation although the same driving forces are used to form amyloid fibrils.

### Inactivating potential hazards of amyloid fibrils by depolymerization

Considering the potential risks caused by amyloid fibrils, various methodologies have been proposed to reduce potential hazards of amyloid fibrils (21, 22, 23, 24, 25). Meanwhile, the matured fibrils may not be the major toxic species. Breaking or disassembly of preformed fibrils can induce toxicity, and CurDAc, a water-soluble curcumin derivative, induced disassembly fibrils and cell toxicity (39). However, the dialysis-related amyloidosis is known to be caused by deposition of amyloid fibrils in joint tissues (8, 40). In addition, understanding the effect of ligands, inhibitors, and salts, *etc*., on protein aggregation is important to better understand the molecular mechanisms that define the pathways of amyloid formation (41), formation of toxic intermediate oligomers (42, 43, 44), and the polymorphism of fibrils (45). Although the effect of strong acid is less physiologically relevant, it will provide deeper insights in understanding of amyloid aggregation.

Amyloid fibrils are an intermolecularly misfolded state formed by breaking the supersaturation barrier (11, 14, 19, 20, 46, 47, 48). Based on this physicochemical principle, it is possible to dissolve preformed fibrils by increasing the solubility above the fibril concentration. In practice, because of the rigid and persistent intermolecular interactions, it may take a long period for dissolution without accelerating agitations such as ultrasonication (49, 50).

We summarize the solvent conditions to stabilize or destabilize amyloid fibrils without modifying the chemical structures (Table S1). Moderate concentrations of anions either derived from salts or strong acids induce amyloid fibrils by decreasing the solubility of denatured proteins and peptides through counter anion binding (15, 17). Decreasing the salt concentration at pI causes pI amyloid formation by hydrophobic and attractive charge–charge interactions (51). In contrast, among various organic solvents, a high concentration (>80%) of dimethyl sulfoxide (DMSO) is useful for dissolving amyloid fibrils (52, 53). DMSO is a polar solvent with a strong potential to become a proton acceptor. Although high concentrations are required, DMSO breaks hydrogen bonds required for amyloid fibril formation. DMSO has been used in clinical treatment (54, 55). Treatment by oral ingestion, direct application to the skin, or intravenous (transdermal) administration improved several clinical outcomes of amyloidosis, such as AL amyloidosis nephrosis, carpal tunnel syndrome, dermal amyloidosis, and gastrointestinal symptoms. However, it is not used anymore as a therapy because of side effects and unclear mechanisms.

The effects of high concentrations of salts depend on whether they are kosmotropic or chaotropic salts. Kosmotropic salts such as ammonium sulfate stabilize amyloid fibrils, and chaotropic salts such as guanidium hydrochloride (GdnHCl) dissolve amyloid fibrils (56). However, when native hen egg white lysozyme at pH 2 is treated with GdnHCl, moderate concentrations of GdnHCl destabilize the native state, leading to the promotion of amyloid fibrils before the GdnHCl-dependent inhibition of amyloid formation (57). These effects are basically independent of the net charge of proteins and the effectiveness follows the Hofmeister series (26, 28).

Other external variables exhibiting consecutive stabilizing and destabilizing effects on amyloid fibrils include fluorinated alcohols such as trifluoroethanol (TFE) or hexafluoroethanol (HFIP) (58), detergents such as SDS (59, 60, 61), temperature (11, 38), and hydrostatic pressure (62, 63, 64, 65) (Table S1). TFE and HFIP are used at high concentrations to dissolve aggregates of the Alzheimer's amyloid peptide Aβ (66, 67, 68) or prion protein peptides (69, 70). However, these alcohols are also known to promote amyloid fibril formation at lower concentrations (58, 71, 72). These observations indicate the importance of the careful recruitment of amyloid-dissolving conditions excluding amyloid-stabilizing conditions.

## Conclusions

Amyloid fibrils are formed when the concentration of precursor peptides or proteins is higher than solubility coupled with the breakdown of supersaturation (11, 20). To dissolve preformed fibrils by changing the solubility, solvent conditions should increase their solubilities so as to exceed the peptide or protein concentrations. Even if a particular condition is effective in increasing the solubility, changing additive conditions may adversely decrease the solubility and promote amyloid formation. Some of the most interesting additives are strong acids. Although strong acids decrease the solution pH markedly below 2, they might strongly promote amyloid formation by the counter anion-binding effects. Any attempts to prevent amyloid formation or to dissolve amyloid fibrils should consider these basic physicochemical principles of protein folding and misfolding. Carefully separating those amyloid-promoting and amyloid-dissolving conditions will reduce the potential risks of amyloid fibrils.

## Experimental procedures

### Materials

β2m was expressed in *Escherichia coli* BL21(DE3) and purified as previously described (73). The purity of the protein solution was confirmed to be more than 95% by SDS-PAGE and MALDI-TOF mass spectroscopy (Bruker Daltonics). Recombinant human insulin was purchased from Roche Diagnostics GmbH, and ThT was from FUJIFILM Wako Pure Chemical Corporation. All other reagents were obtained from Nacalai Tesque.

### Amyloid fibril formation of β2m and insulin

To investigate the effects of NaCl and HCl on β2m amyloid formation, the sample solutions contained 25 μM (0.3 mg/ml) β2m, 5 μM ThT, and various concentrations of NaCl or HCl in the presence of 10 mM HCl. 10 mM HCl was used to adjust the pH of the solution to ∼2.0. For the formation of insulin amyloid fibrils, the sample solutions contained 1 mg/ml insulin, 5 μM ThT, 10 mM HCl, and various concentrations of NaCl or HCl.

To compare the effects of Na_2_SO_4_ and H_2_SO_4_, the sample solutions contained 25 μM β2m, 5 μM ThT, various concentrations of Na_2_SO_4_ or H_2_SO_4_, and 10 mM HCl, since 10 mM SO_4_^−^ has the potential to induce amyloid formation by itself (26). For amyloid formation using trichloroacetic acid (TCA) or formic acid (HCOOH), the sample solutions contained 25 μM β2m, 5 μM ThT, 10 mM HCl, and various concentrations of TCA or formic acid.

Amyloid formation was performed with a Hitachi F7000 spectrofluorometer, in which the sample solution of 2 ml, as described above, in a glass cuvette was irradiated with ultrasonic pulses from the ultrasonic generator tightly attached to the sidewall of the cuvette (Elekon Science Co) (27). A cycle involving 1 min of ultrasonication and 9 min of quiescence was repeated, and the solution was stirred with a stirring magnet at 600 rpm at 37 °C. The frequency and power of ultrasonic pulses were 27.5 kHz and 0.14 W, respectively. Fluorescence emission spectra from 430 to 550 nm were measured repeatedly with excitation at 445 nm, and 90° LS at 448 nm and ThT fluorescence at 485 nm were plotted. For the ThT assay under neutral conditions, an aliquot of 20 μl was taken from each reaction tube after the amyloid formation and mixed with 2 ml of 5 μM ThT in 50 mM sodium phosphate buffer (pH 7.5). The ThT fluorescence was immediately measured using a Hitachi F7000 spectrofluorometer with excitation at 445 nm and emission at 485 nm.

### CD measurement

Far-UV CD spectra were measured with a Jasco spectropolarimeter J-820 (Jasco Co, Ltd) using a quartz cell with a light path of 1 mm and protein concentration of 0.15 mg/ml at 25 °C. The results are expressed as the mean residue ellipticity [*θ*] (deg cm^2^ dmol^−1^).

### TEM observation

A 5-μl aliquot of the sample solution was placed on a collodion-coated copper grid (Nisshin EM Co) for 1 min, and excess solution was removed by blotting with filter paper. The grid was negatively stained with a 5-μl droplet of 2% (w/v) phosphotungstic acid for 1 min. The liquid on the grid was removed by blotting and then dried. TEM was performed using a H-7650 transmission electron microscope (HITACHI) operating at an acceleration voltage of 80 kV.

## Data availability

All data are contained within the manuscript.

## Supporting information

This article contains supporting information.

## Conflict of interest

The authors declare that they have no conflicts of interest with the contents of this article.
