# Supplementary material for: Strong acids induce amyloid fibril formation of β2-microglobulin via an anion-binding mechanism
Source: J Biol Chem. 2021 Oct 7;297(5):101286. doi: 10.1016/j.jbc.2021.101286 (PMC8564678; doi:10.1016/j.jbc.2021.101286)
Supplement: Figures S1–S9 and Table S1 [file mmc1.pdf]

Supplementary Information for

Strong acids induce amyloid fibril formation of β_2-microglobulin via an anion-binding mechanism

Keiichi Yamaguchi, Kenshiro Hasuo, Masatomo So, Kensuke Ikenaka, Hideki Mochizuki, and Yuji Goto

Yuji Goto

E–mail: gtyj8126@protein.osaka-u.ac.jp

This PDF file includes:

Table S1
Figures S1 to S9
References

Table S1. Effects of solvent conditions on amyloid formation and underlying mechanisms on the basis of the solubility mechanism.

**Solvent conditions**	**Amyloid formation**	**Amyloid dissolution**	**Comments and references**
Solvent-dependent amyloid formation or dissolution			
Moderate concentrations of anions at pH below 2	Yes, by decreased solubility through counter ion binding	Not observed	The effectiveness of anions follows electroselectivitiy series (1,2).
Low salt concentrations at pI	Yes, by pI precipitation through hydrophobic and attractive charge-charge interactions	Not observed	The effects were clearly observed for α-synuclein at pI (=4.7) (3).
DMSO	Not observed	Yes, by increased solubility at high DMSO concentrations (>80%).	The most effective solvent to dissolve amyloid fibrils by breaking hydrogen bonds (4,5).
High concentrations of salts or ions at various pHs	Yes, by decreased solubility by Kosmotropic effects (e.g., sulfate and polyphosphates)	Yes, by increased solubility by Caotropic effects (e.g., guanidium anion)	The effects are independent of the net charge of proteins and the effectiveness follows the Hofmeister series (1,6).
Solvent-dependent amyloid formation and dissolution depending on the concentration			
Fluorinated alcohols (e.g., TFE, HFIP)	Yes, by decreased solubility at moderate concentrations	Yes, by increased solubility at high concentrations	The decrease in solubility is caused by the formation of hydrophobic clusters of alcohols (7).
Detergents (e.g., SDS)	Yes, by decreased solubility at concentrations near CMC	Yes, by increased solubility above CMC	The mechanisms resemble those of alcohols (8).
Temperature	Yes, by decreased solubility at 40-80 °C, in particular, when coupled with protein heat-denaturation	Yes, by increased solubility at low and very high temperatures	The mechanisms resemble those of cold- and heat-denaturation of globular proteins (9,10).
Hydrostatic pressure	Yes, by decreased solubility when coupled with pressure-denaturation	Yes, by smaller partial specific volume of depolymerized monomers than that of amyloid fibrils	(11-14)

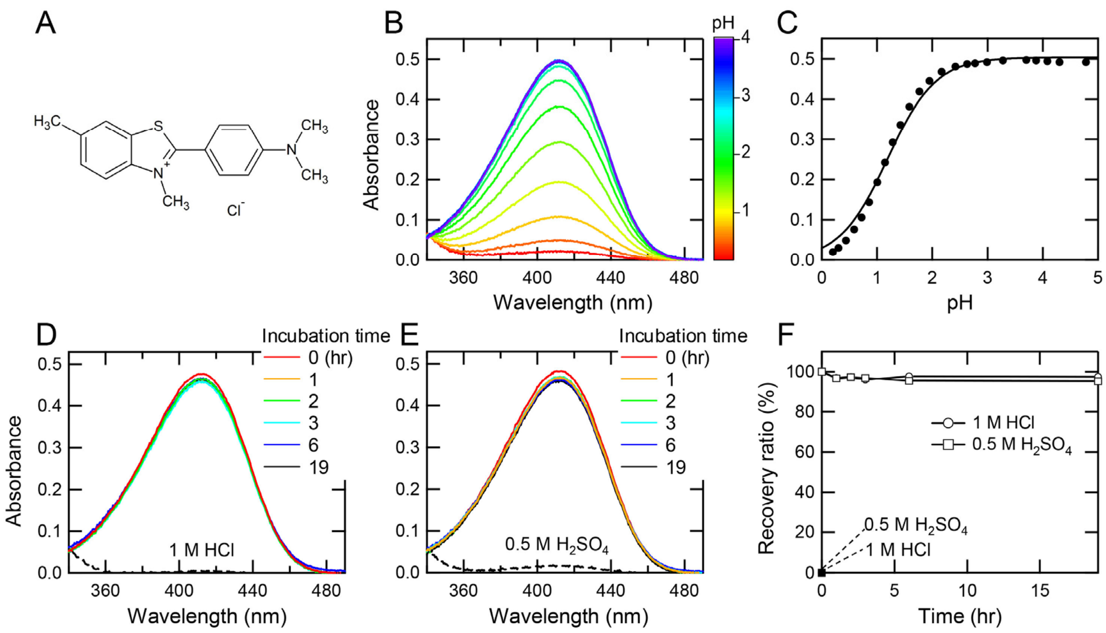

Figure S1. Absorption spectra and stabilities of ThT at acidic pH. (A) Chemical structure of ThT. (B) Absorption spectra of ThT at varying concentrations of HCl and 20 μM ThT at 25 °C. The pH of the solution is shown by a color bar on the right. (C) ThT absorbance at 412 nm plotted against the solution pH. Curve fitting was performed using an equation for the acid dissociation constant (pK_a), and a pK_a value of 1.2 was obtained. (D, E) The absorption spectra of ThT at pH 7 after incubation in 1.0 M HCl (D) and 0.5 M H_2SO_4 (E) at 37 °C. Incubation time is shown in the figures. (F) Recovery ratio of ThT after incubation in 1.0 M HCl (open circles) or 0.5 M H_2SO_4 (open squares). Recovery ratio was calculated form the intensity at 412 nm in the absorption spectra. The absorption of ThT in 1.0 M HCl or 0.5 M H_2SO_4 was zero as indicated.

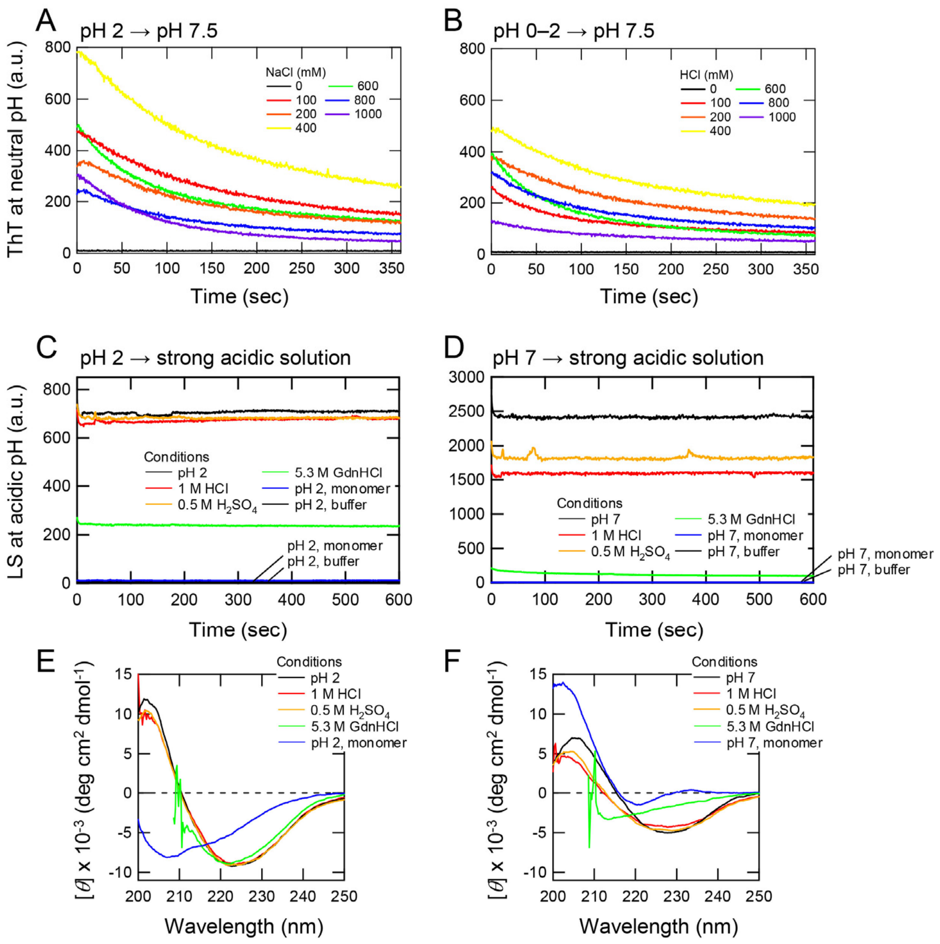

Figure S2. Destabilization of performed amyloid fibrils after pH-jump. (A, B) ThT fluorescence of amyloid fibrils formed at 10 mM HCl including varying concentrations of NaCl (A) and HCl (B) after pH-jump from acidic to neutral pH conditions. (C, D) LS intensities and (E, F) CD spectra after transferring amyloid fibrils formed at pH 2 (C, E) and pH 7 (D, F) to strong acidic solution. For the LS experiments, the wavelengths for excitation and emission were both set at 445 nm. The solution conditions including 5.3 M GdnHCl were also shown in the figures. After transferring amyloid fibrils formed at pH 2 to 1 M HCl or 0.5 M H_2SO_4, both of their LS intensities and CD spectra changed little compared to those transferring into 5.3 M GdnHCl, indicating that preformed amyloid fibrils at pH 2 were not destabilized by acid treatment. Meanwhile, after transferring amyloid fibrils formed at pH 7 to 1 M HCl or 0.5 M H_2SO_4, their LS intensities, not CD, decreased compared to those transferring into 5.3 M GdnHCl. These results suggest that preformed amyloid fibrils at pH 7 are hardly destabilized by strong acid treatment, since the cross-β-sheet structure monitored by CD remains almost unchanged.

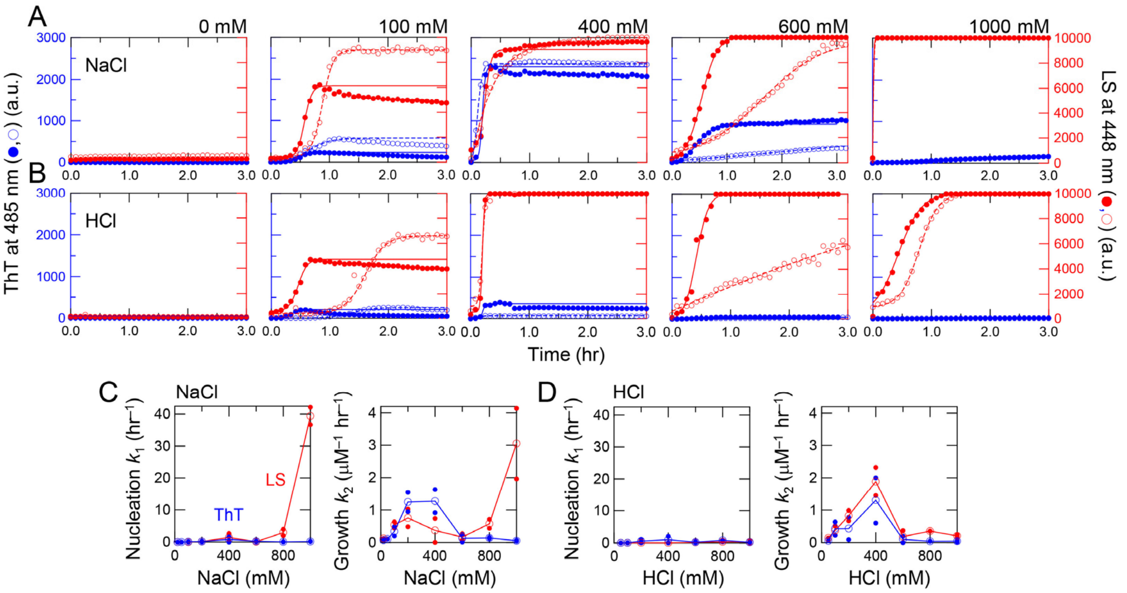

Figure S3. Kinetic analysis of amyloid formation of β2m at varying concentrations of NaCl and HCl. (A, B) Kinetics of amyloid formation monitored by ThT fluorescence (blue) and LS (red), taken from figure 1A, B. The kinetics data were analyzed using the Finke–Watzky (F–W) 2-step kinetics model (15,16).

$$[\mathrm{B}]_t = [\mathrm{A}]_0 - \frac{\frac{k_1}{k_2} + [\mathrm{A}]_0}{1 + \frac{k_1}{k_2[\mathrm{A}]_0}\exp(k_1 + k_2[\mathrm{A}]_0)t}$$

where $[\mathrm{B}]_t$ represents the concentration of aggregated protein at time t, $[\mathrm{A}]_0$ represents the initial protein concentration, and k_1 and k_2 are the rate constants for nucleation and growth, respectively. The curve fitting was not performed for both ThT fluorescence and LS at 0 mM NaCl and HCl and, it was not performed for ThT fluorescence at 1000 mM HCl. (C, D) After curve fitting of the kinetics data, the rate constants for their nucleation k_1 and growth k_2 values were obtained at varying concentrations of NaCl (C) and HCl (D). Since the ThT fluorescence monitored the formation of amyloid fibrils, the increases in k_2 were observed at the concentrations of 400 mM NaCl and HCl. On the other hand, the rate constants for both k_1 and k_2 estimated from LS increased significantly at the concentrations of 800–1000 mM NaCl, indicating the formation of amorphous aggregates. Although the ThT fluorescence was quenched at higher concentrations of HCl, the rate constants for both k_1 and k_2 estimated from LS did not increase at 1000 mM HCl. The differences in solution pH and sodium ion may affect the kinetics of amorphous aggregation.

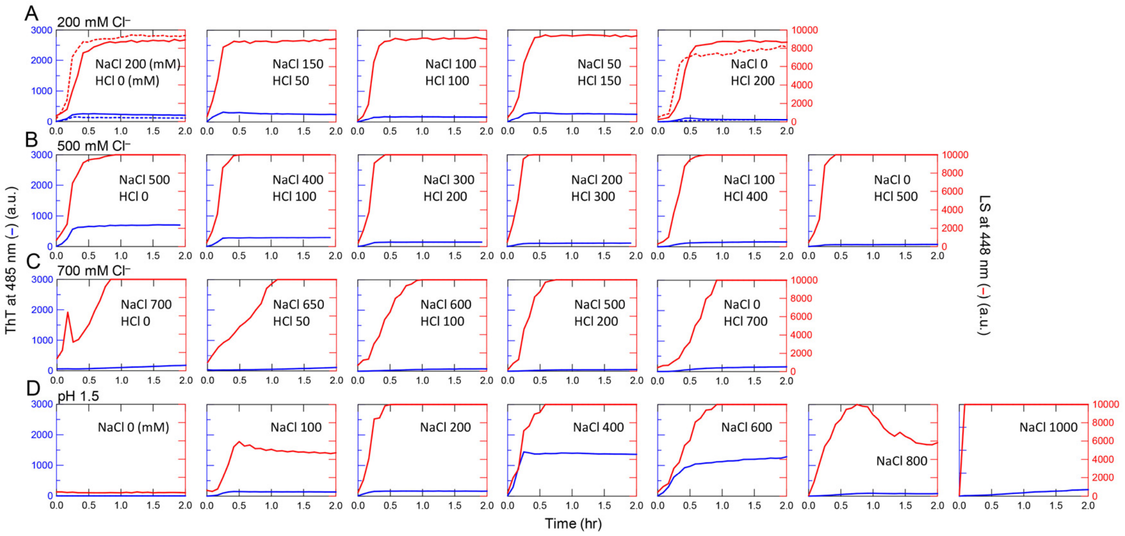

Figure S4. Amyloid formation of β2m at varying concentrations of NaCl and HCl under ultrasonication. (A–D) Kinetics of amyloid formation monitored by ThT fluorescence (blue) and LS (red) at 200 (A), 500 (B), 700 mM Cl^- (C), and pH 1.5 (D) in the presence of various concentration of NaCl and HCl. Solid lines and dashed lines show separate measurements at 200 mM Cl^-, while most of experiments were performed only once. This is because we wanted to perform the measurements are different solvent conditions as many as possible to construct the phase diagram. The concentrations of NaCl and HCl are shown in the figures, where all solutions contained 10 mM HCl and the Cl^- concentrations indicated do not include the contribution of 10 mM HCl.

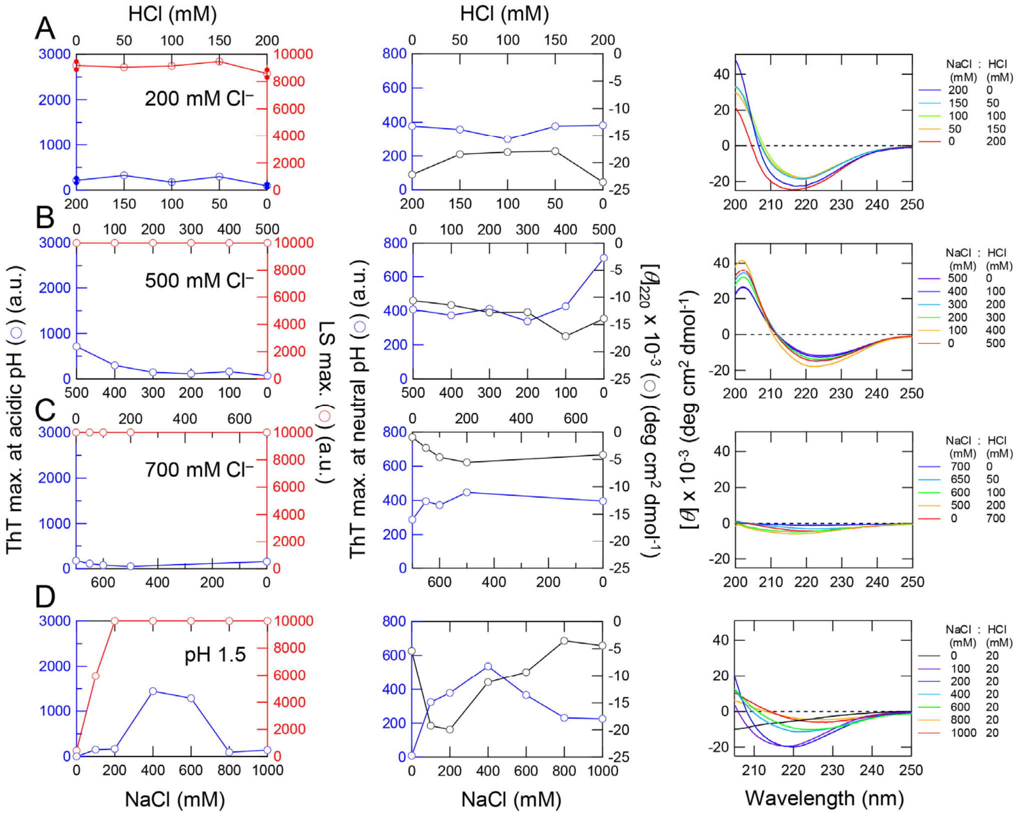

Figure S5. Amyloid formation at varying concentrations of NaCl and HCl. Total Cl^{-} concentrations were 200 (A), 500 (B), 700 mM (C), and pH 1.5 (D). Left column: Maximum values of ThT fluorescence (blue) and LS (red) at an acidic pH. Middle column: Maximum values of ThT fluorescence under neutral pH conditions (blue) and CD ellipticities at 220 nm (black). Right column: CD spectra after amyloid formation. All solutions contained 10 mM HCl and the Cl^{-} concentrations indicated do not include the contribution of 10 mM HCl.

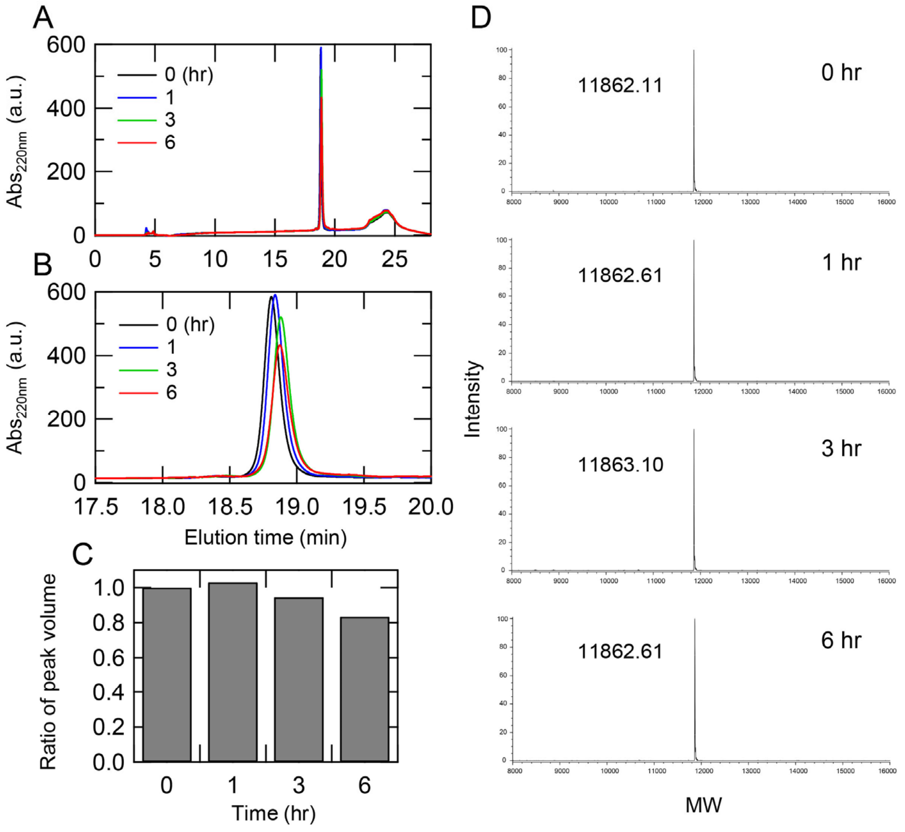

Figure S6. HPLC and ESI-Mass analyses of β2m at 500 mM H_2SO_4 and 37 °C under ultrasonication. (A, B) HPLC profiles of β2m in the presence of 500 mM H_2SO_4 after incubation for 0 (black), 1 (blue), 3 (green), and 6 hrs (red) (A). (B) is an enlarged image of (A). (C) Relative peak volume of β2m in the presence of 500 mM H_2SO_4 after incubation for 0, 1, 3, and 6 hrs. (D) ESI-Mass spectra of β2m fractions in the presence of 500 mM H_2SO_4 after incubation for 0, 1, 3, and 6 hrs. The theoretical molecular weight of β2m is 11862.

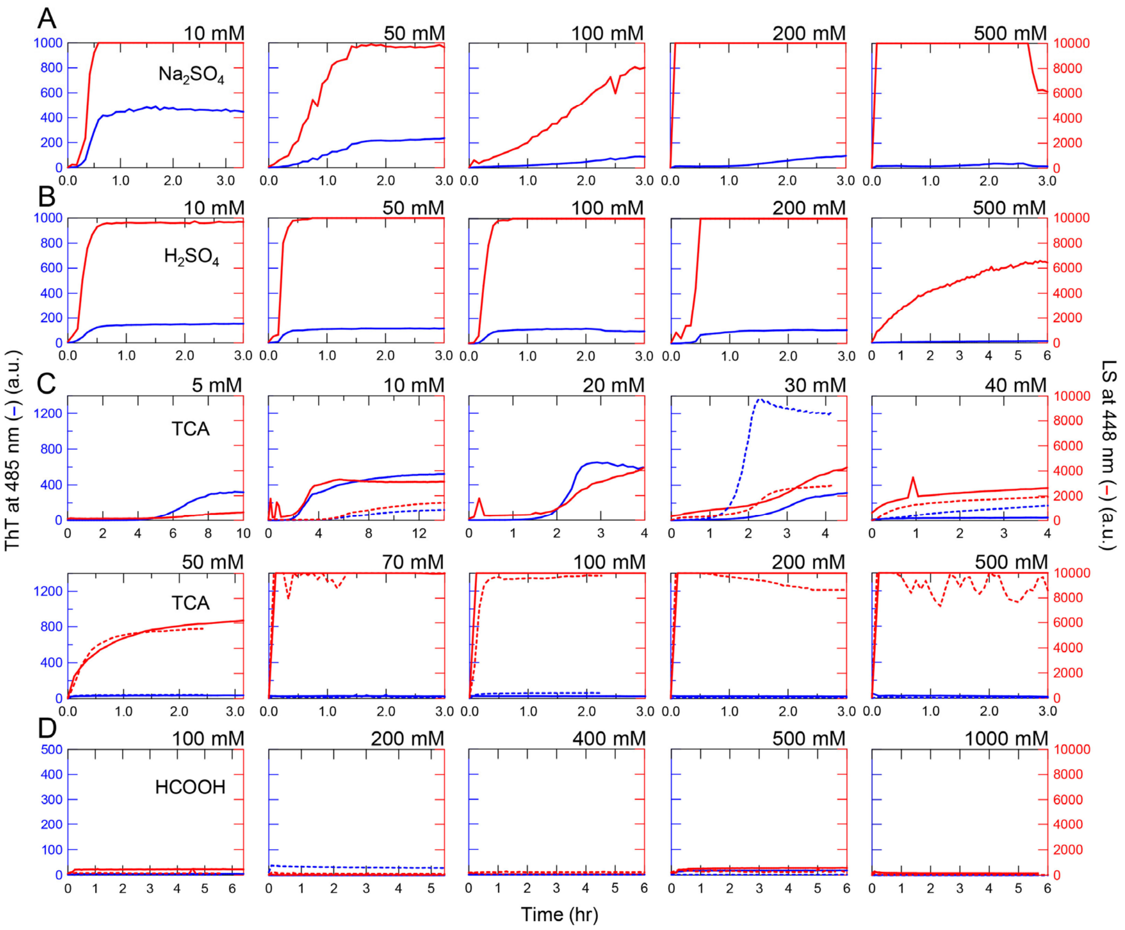

Figure S7. Amyloid formation of β2m at varying concentrations of Na_2SO_4, H_2SO_4, TCA, and formic acid. (A–D) Kinetics of amyloid formation monitored by ThT fluorescence (blue) and LS (red) at varying concentrations of Na_2SO_4 (A), H_2SO_4 (B), TCA (C), and formic acid (D) in the presence of 10 mM HCl under ultrasonication. Solid lines and dashed lines show separate measurements under the same conditions.

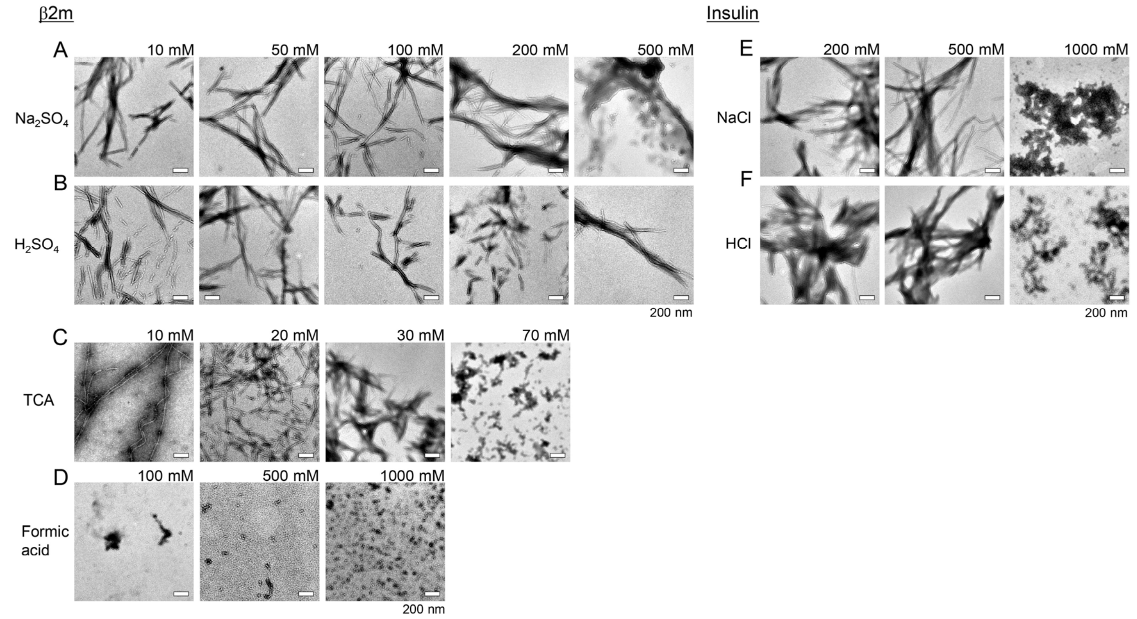

Figure S8. EM images of β2m and insulin after amyloid formation. EM images of (A–D) β2m aggregates at varying concentrations of Na_2SO_4 (A), H_2SO_4 (B), TCA (C), and formic acid (D), and (E, F) insulin aggregates at varying concentrations of NaCl (E) and HCl (F) in the presence of 10 mM HCl. The scale bars are 200 nm. The HCl concentrations in (F) do not include the 10 mM HCl present in all samples.

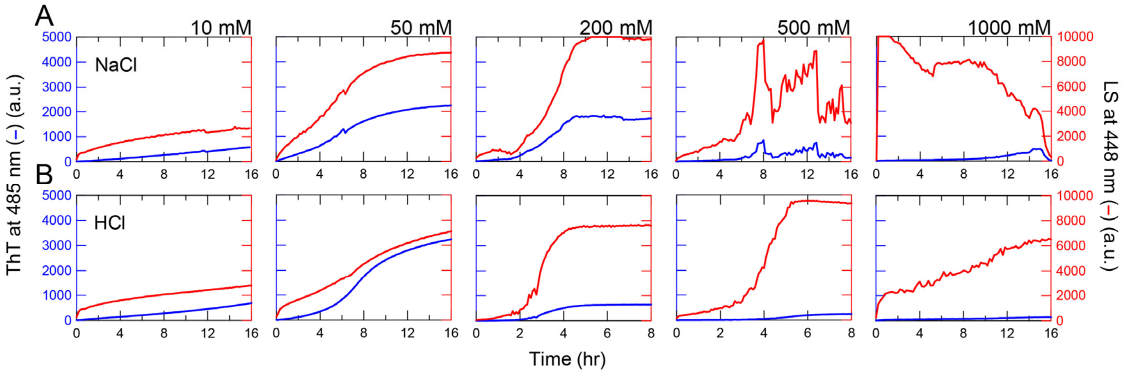

Figure S9. Amyloid formation of insulin. (A, B) Kinetics of amyloid formation monitored by ThT fluorescence (blue) and LS (red) at varying concentrations of NaCl (A) and HCl (B) in the presence of 10 mM HCl under ultrasonication. The HCl concentrations in (B) do not include the contribution of 10 mM HCl present in all samples.

References

1. Raman, B., Chatani, E., Kihara, M., Ban, T., Sakai, M., Hasegawa, K., Naiki, H., Rao Ch, M., and Goto, Y. (2005) Critical balance of electrostatic and hydrophobic interactions is required for β2-microglobulin amyloid fibril growth and stability. *Biochemistry* **44**, 1288-1299
2. Goto, Y., Adachi, M., Muta, H., and So, M. (2018) Salt-induced formations of partially folded intermediates and amyloid fibrils suggests a common underlying mechanism. *Biophys Rev* **10**, 493-502
3. Furukawa, K., Aguirre, C., So, M., Sasahara, K., Miyanoiri, Y., Sakurai, K., Yamaguchi, K., Ikenaka, K., Mochizuki, H., Kardos, J., Kawata, Y., and Goto, Y. (2020) Isoelectric point-amyloid formation of α-synuclein extends the solubility and supersaturation-limited mechanism. *Curr. Res. Struct. Biol.* **2**, 35-44.
4. Hoshino, M., Katou, H., Hagihara, Y., Hasegawa, K., Naiki, H., and Goto, Y. (2002) Mapping the core of the β2-microglobulin amyloid fibril by H/D exchange. *Nat Struct Biol* **9**, 332-336
5. Hirota-Nakaoka, N., Hasegawa, K., Naiki, H., and Goto, Y. (2003) Dissolution of β2-microglobulin amyloid fibrils by dimethylsulfoxide. *J Biochem* **134**, 159-164
6. Zhang, C. M., Yamaguchi, K., So, M., Sasahara, K., Ito, T., Yamamoto, S., Narita, I., Kardos, J., Naiki, H., and Goto, Y. (2019) Possible mechanisms of polyphosphate-induced amyloid fibril formation of β2-microglobulin. *Proc Natl Acad Sci U S A* **116**, 12833-12838
7. Yamaguchi, K., Naiki, H., and Goto, Y. (2006) Mechanism by which the amyloid-like fibrils of a β2-microglobulin fragment are induced by fluorine-substituted alcohols. *J Mol Biol* **363**, 279-288
8. So, M., Ishii, A., Hata, Y., Yagi, H., Naiki, H., and Goto, Y. (2015) Supersaturation-limited and unlimited phase spaces compete to produce maximal amyloid fibrillation near the critical micelle concentration of sodium dodecyl sulfate. *Langmuir* **31**, 9973-9982
9. Noji, M., Sasahara, K., Yamaguchi, K., So, M., Sakurai, K., Kardos, J., Naiki, H., and Goto, Y. (2019) Heating during agitation of β2-microglobulin reveals that supersaturation breakdown is required for amyloid fibril formation at neutral pH. *J Biol Chem* **294**, 15826-15835
10. Noji, M., Samejima, T., Yamaguchi, K., So, M., Yuzu, K., Chatani, E., Akazawa-Ogawa, Y., Hagihara, Y., Kawata, Y., Ikenaka, K., Mochizuki, H., Kardos, J., Otzen, D. E., Bellotti, V., Buchner, J., and Goto, Y. (2021) Breakdown of supersaturation barrier links protein folding to amyloid formation. *Commun Biol* **4**, 120
11. Lee, Y. H., Chatani, E., Sasahara, K., Naiki, H., and Goto, Y. (2009) A comprehensive model for packing and hydration for amyloid fibrils of β2-microglobulin. *J Biol Chem* **284**, 2169-2175
12. Shah, B. R., Maeno, A., Matsuo, H., Tachibana, H., and Akasaka, K. (2012) Pressure-accelerated dissociation of amyloid fibrils in wild-type hen lysozyme. *Biophys J* **102**, 121-126
13. Luong, T. Q., Erwin, N., Neumann, M., Schmidt, A., Loos, C., Schmidt, V., Fandrich, M., and Winter, R. (2016) Hydrostatic Pressure Increases the Catalytic Activity of Amyloid Fibril Enzymes. *Angew Chem Int Ed Engl* **55**, 12412-12416

14. Jaworek, M. W., Schuabb, V., and Winter, R. (2018) Pressure and cosolvent modulation of the catalytic activity of amyloid fibrils. *Chem Commun (Camb)* **54**, 5696-5699
15. Morris, A. M., Watzky, M. A., Agar, J. N., and Finke, R. G. (2008) Fitting neurological protein aggregation kinetic data via a 2-step, minimal/"Ockham's razor" model: the Finke-Watzky mechanism of nucleation followed by autocatalytic surface growth. *Biochemistry* **47**, 2413-2427
16. Watzky, M. A., Morris, A. M., Ross, E. D., and Finke, R. G. (2008) Fitting yeast and mammalian prion aggregation kinetic data with the Finke-Watzky two-step model of nucleation and autocatalytic growth. *Biochemistry* **47**, 10790-10800
